# Efficacy of Robot-Assisted Ureteroureterostomy in Patients with Complex Ureteral Stricture after Ureteroscopic Lithotripsy

**DOI:** 10.3390/jcm12247726

**Published:** 2023-12-16

**Authors:** Shuzo Hamamoto, Kazumi Taguchi, Kengo Kawase, Rei Unno, Masahiko Isogai, Koei Torii, Shoichiro Iwatsuki, Toshiki Etani, Taku Naiki, Atsushi Okada, Takahiro Yasui

**Affiliations:** Department of Nephro-Urology, Nagoya City University Graduate School of Medical Sciences, Nagoya 4678601, Japan; ktaguchi@med.nagoya-cu.ac.jp (K.T.); kawase@med.nagoya-cu.ac.jp (K.K.); unno@med.nagoya-cu.ac.jp (R.U.); masa22@med.nagoya-cu.ac.jp (M.I.); koei0624@med.nagoya-cu.ac.jp (K.T.); iwatsuki@med.nagoya-cu.ac.jp (S.I.); uroetani@med.nagoya-cu.ac.jp (T.E.); naiki@med.nagoya-cu.ac.jp (T.N.); a-okada@med.nagoya-cu.ac.jp (A.O.); yasui@med.nagoya-cu.ac.jp (T.Y.)

**Keywords:** robot-assisted ureteroureterostomy, ureteral stricture, ureteroscopic lithotripsy, microcalcification

## Abstract

Background: Ureteral stricture (US) postureteroscopic lithotripsy (URSL) has emerged as a severe complication with the widespread use of laser technology. Furthermore, managing a complex US is challenging. Therefore, this study evaluated the efficacy of robot-assisted ureteroureterostomy (RAUU) in addressing US post-URSL and analyzed the pathology of transected ureteral tissues to identify the risk factors for US. Methods: we conducted a prospective cohort study on patients who underwent RAUU for URSL-induced US from April 2021 to May 2023. Results: A total of 14 patients with a mean age of 49.8 years were included in this study. The mean stricture length on radiography was 22.66 ± 7.38 mm. Nine (64.2%) patients had experienced failure with previous interventions. The overall success rate was 92.9%, both clinically and radiographically, without major complications, at a mean follow-up of 12.8 months. The pathological findings revealed microcalcifications and a loss of ureteral mucosa in 57.1% and 28.6% of patients, respectively. Conclusions: The RAUU technique shows promise as a viable option for US post-URSL in appropriately selected patients despite severe pathological changes in the ureter. Therefore, the migration of microcalcifications to the site of ureteral perforation may be a significant factor contributing to US development.

## 1. Introduction

The use of ureteroscopic lithotripsy (URSL), a minimally invasive technique for managing urinary calculi, has steadily increased over the past few decades [1,2]. Despite the procedure’s efficacy in stone removal, it may lead to severe postoperative complications, including sepsis, urinary injury, and ureteral stricture (US). Notably, the incidence of US post-URSL has gradually increased to approximately 3.0% with the advances in laser technology [3,4]. Therefore, the prompt treatment of these strictures is critical since they can lead to significant issues, such as pain, hydronephrosis, recurrent urinary tract infections, and impaired renal function if left untreated.

Nevertheless, managing US remains challenging. Endoscopic management, which involves the combination of laser incision and balloon dilation, is the first treatment option for US with a length of <2 cm [5]. Razdan et al. reported a success rate of endoscopic management as high as 74% [5]. However, endoscopic treatment, considered the least-invasive option, may have reduced success rates when URSL is the underlying cause of US [6]. Traditional surgical techniques, including open ureteral reimplantation and laparoscopic ureteral reconstruction, have been employed to manage complex or recurrent US [7,8], although they have inherent limitations, including extended hospital stays, increased postoperative pain, and prolonged recovery periods [9,10]. Consequently, robot-assisted ureteral reconstruction has emerged as a viable option with favorable outcomes because of the advantages of three-dimensional vision, magnified visibility, and adjunct near-infrared fluorescence (NIRF) imaging [9,10,11,12].

Despite these promising outcomes, the specific utility of robot-assisted techniques for managing US post-URSL remains underexplored. Therefore, this study aimed to prospectively evaluate the efficacy of robot-assisted ureteroureterostomy (RAUU) against US post-URSL and analyze the pathology of transected ureteral tissues to identify potential risk factors for US during URSL.

## 2. Materials and Methods

### 2.1. Patient Population

We included 14 patients who underwent RAUU for US post-URSL at Nagoya City University Hospital from April 2021 to May 2023. This prospective cohort study was approved by the Institutional Review Board of Nagoya City University Graduate School of Medical Sciences (approval number: 46-21-0004), and it adhered to the guidelines of the Declaration of Helsinki (revised in 2013). Informed consent was obtained from all participants.

Patients were selected for surgery based on the evidence of strictures on diagnostic imaging. The inclusion criteria were patients who had hydronephrosis (grade > 2 according to the Ellenbongen classification [13]) due to US post-URSL. In this study, US was defined as at least one of the following: the presence of long-segment US (2–4 cm) confirmed through a radiographic evaluation after failed management, with the deterioration of kidney function on the affected side (<20% diagnosed using renal scintigraphy).

Patients who were aged <19 years, undergoing treatment for malignant disease, had recent pyelonephritis, underwent dialysis treatment, could not undergo general anesthesia, or had suspected pregnancies were excluded from this study.

### 2.2. Data Collection

Clinical and imaging data were prospectively collected, encompassing patient demographics, including previous stone characteristics that were treated by using URSL and preoperative imaging, such as ultrasound, computed tomography (CT), retrograde ureterography, and renal scintigraphy. Stone size was defined as the longest diameter observed on the preoperative imaging, whereas stone volume was calculated by using Tiselius’s formula (length × width × 3.14 × 0.25) [14]. US characteristics, including location, site, length, and previous management, were also evaluated. Specifically, the US length was measured by using antegrade or retrograde ureterographic imaging.

This study’s primary outcome was to evaluate the success and complication rates of this procedure. The success status was determined by the absence of symptoms without a ureteral stent and nephrostomy, relief of hydronephrosis on ultrasonography and CT 3 months postoperatively, and no US recurrence at the latest follow-up. All patients were evaluated for perioperative complications according to the modified Clavien–Dindo classification [15]. Secondary outcomes were the total surgical duration, console time, blood loss during surgery, and pathological characteristics of the stricture sites. A histopathological analysis was performed by using hematoxylin and eosin staining and Masson’s trichrome staining to determine the tissue levels of fibrosis, and Pizzolato staining [16] and polarized light microscopy (using a BX51-33-O instrument; Olympus, Tokyo, Japan) were used to observe calcification at the US site.

### 2.3. Surgical Technique

#### Patient and Robot Setup

**Figure 1:**
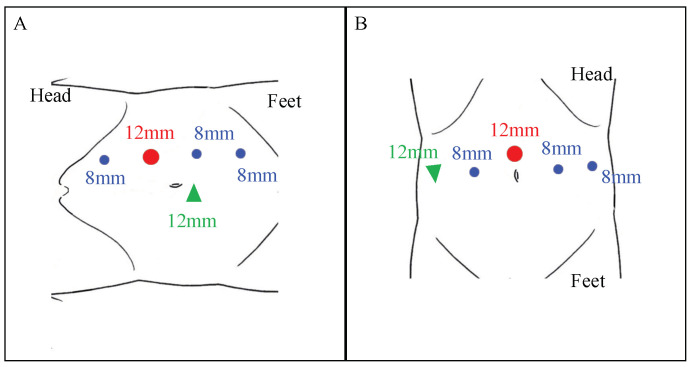
(**A**) Surgical position during left robot-assisted ureteroureterostomy for proximal and middle ureteral strictures. (**B**) Surgical position for distal ureteral strictures. Red circle: a camera port. Green triangle: assistant port. Blue circle: robotic ports for the instrument.

Two surgeons (SH and KT) who have conducted >200 robotic surgeries performed all the surgeries transperitoneally by using the da Vinci Xi surgical system (Intuitive Surgical, Sunnyvale, CA, USA). Under general anesthesia, before the RAUU, an open-end ureteral or illumination catheter (infrared illumination system (IRIS); Stryker, Kalamazoo, MI, USA) was inserted transurethrally and positioned immediately below the stricture site under fluoroscopic guidance in the lithotomy position. The patient positioning and port placement were modified according to the US location. Patients undergoing proximal and middle US were positioned in the lateral flank position, with the affected site facing upwards. Generally, we employed four robotic trocars and one 12 mm trocar for assistance. The 12 mm camera port was positioned periumbilically at the outer edge of the abdominal rectus muscle, and 8 mm working ports were placed 7 cm laterally to each side of the camera port in a straight line (Figure 1A). In contrast, patients who underwent distal US were positioned in the low-lithotomy position with a steep Trendelenburg. A camera port was fixed above the umbilicus, and four trocars were used as the working ports, including a 12 mm right lateral assistant port.

### 2.4. Stricture Identification

**Figure 2:**
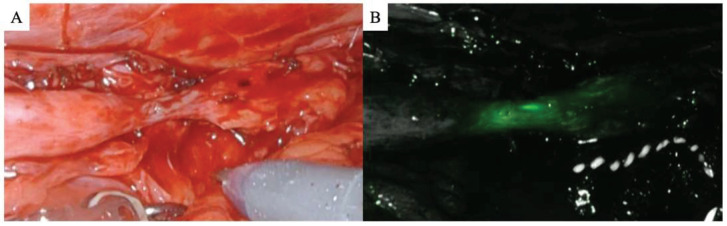
Stricture identification using retrograde indocyanine green (ICG) injection in a patient with left ureteral stricture (**A**) in the absence of near-infrared fluorescence (NIRF) and (**B**) under NIRF. ICG could not be visualized at the proximal side of the ureteral stricture due to complete obstruction.

**Figure 3:**
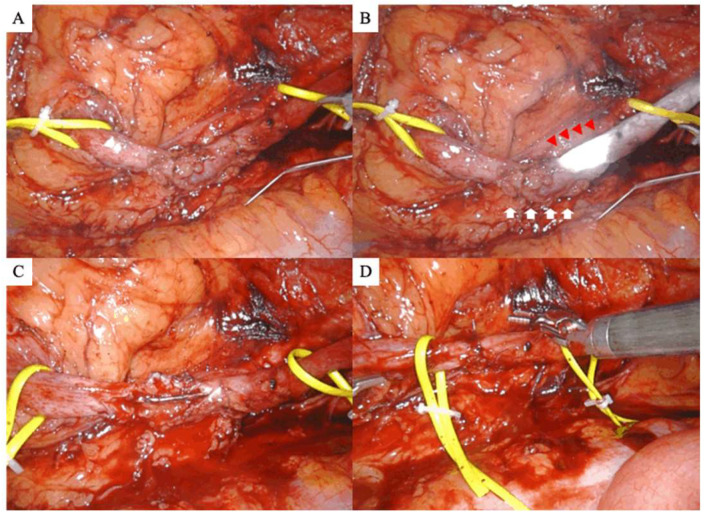
Robot-assisted ureteroureterostomy in a patient with left ureteral stricture. Identification of ureteral stricture (**A**) in the absence of an infrared illumination system (IRIS) and (**B**) under an IRIS catheter. Red and white arrowheads indicate the top of the IRIS catheter and the narrow segment of the ureter, respectively. (**C**) Excision of the stricture segment. (**D**) End-to-end anastomosis of the healthy ureters.

Following medial mobilization of the colon’s affected side, the retroperitoneal space was expanded to locate the ureter. The US location was identified in some cases by using an intraureteral injection of indocyanine green (ICG) under NIRF, as previously described [11] (Figure 2A,B). Next, 25 mg of ICG was prepared in 10 mL of distilled water and injected retrogradely through an open-end ureteral catheter. However, illumination with an IRIS catheter was used in other cases to identify and secure the stricture (Figure 3A,B).

### 2.5. Dissection of the Ureteral Stricture, Anastomosis, and Stenting

The narrow segment was incised longitudinally on the ventral side using Potts scissors (Intuitive Surgical, Sunnyvale, CA, USA) after the length of the US was confirmed (Figure 3C). Two stitches were sutured between the proximal and distal ends of the healthy ureter before the ureter was fully transected to prevent misleading the anastomotic direction. After inserting a 6-Fr double-J ureteral catheter, the segmental lesion was completely resected, and anastomosis was performed by using 4–0 Monocryl sutures (Figure 3D). Finally, a suction drain was placed around the reconstruction site after the procedure, and ureteral stents were removed 6 weeks postoperatively by using retrograde pyelography to evaluate the anastomosis site’s status and resolution of obstruction.

### 2.6. Statistical Analysis

All data were analyzed by using EZR software for R (The R Foundation for Statistical Computing, Vienna, Austria) [17]. The quantitative variables were expressed as the mean ± standard deviation or median (interquartile ranges (IQR)), depending on the distribution pattern. Categorical data were expressed as numbers (percentages).

## 3. Results

### 3.1. Clinical Characteristics

Table 1 presents the demographic and clinical characteristics of the 14 patients with previous urinary calculi. The cohort comprised 10 males and 4 females, with a mean age of 49.8 years. Additionally, the urinary calculi that resulted in US were distributed in the ureteropelvic junction (UPJ) (28.6%), proximal ureter (50.0%), middle ureter (14.3%), and distal ureter (7.1%), and the median stone volume was 78.11 (IQR, 72.76–99.11) mm^2^. All patients underwent URSL, with a median of 2.00 (IQR, 1.00–2.00) procedures performed.

Table 2 presents the details of the US. The US distribution sites encompassed the UPJ (21.4%), proximal ureter (50.0%), middle ureter (21.4%), and distal ureter (7.1%). Notably, four (28.6%) patients exhibited a mismatch between the location of the urinary calculi and US. The mean stricture length on radiography was 22.66 ± 7.38 mm. Nine (64.2%) patients had undergone previously failed interventions, including eight who underwent endoscopic incision and balloon dilation. Regarding the grade of preoperative hydronephrosis, 4 (28.6%) and 10 (71.4%) patients had moderate and severe hydronephrosis, respectively. Nephrostomy was inserted in 3 (21.4%) patients, while 10 (71.4%) had a ureteral stent inserted before surgery.

### 3.2. Surgical Outcomes

Table 3 presents the perioperative and follow-up outcomes. All robotic procedures were completed successfully without intraoperative complications or the need for conversion. ICG was injected intraureterally in eight (57.1%) patients under NIRF to identify the US, while an illuminating IRIS catheter was used in six (42.9%). Regarding the intraoperative variables, the mean console time was 164.36 ± 44.18 min, and the median blood loss was 10.00 (IQR, 0.00–57.00) mL.

Postoperatively, two patients experienced complications: one patient had a port-site infection requiring antibiotic treatment, and the other reported postoperative pain after discharge. However, no major complications occurred (more than grade III according to the Clavien–Dindo classification). The ureteral stents were successfully removed in all patients, and postoperative hydronephrosis improved or disappeared in 13 patients at 3 months postoperation. Among the 13 patients in whom preoperative hydronephrosis was observed, 3 (21.4%) had no hydronephrosis, and 10 (71.4%) exhibited mild hydronephrosis without clinical significance. Furthermore, the overall success rate, both clinically and radiographically, was 92.9% at a mean follow-up of 12.8 months.

### 3.3. Pathological Analysis

**Figure 4:**
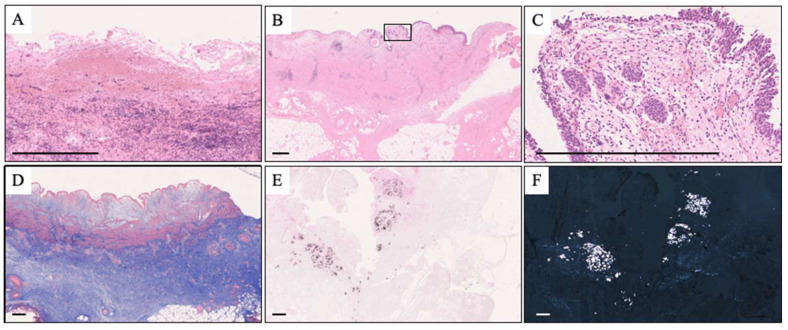
Pathological findings of the transected ureters. (**A**–**C**) Hematoxylin and eosin staining of the tissues. Loss of ureteral mucosa and inflammatory cell infiltration are observed in the tissue (**A**,**B**). (**C**) The high-power magnification of the square in Figure 2B and foreign-body giant cells can be observed in the ureteral submucosa. (**D**) Masson’s trichrome stain shows the fibrosis of the periureteral tissues. (**E**) Pizzolato stain and (**F**) polarized light microscope indicate the existence of microcalcification (scale bar: 500 μm).

The transected ureter’s mean length was 26.36 ± 39.34 mm, and hematoxylin and eosin staining (Figure 4A–C) revealed the loss of the ureteral mucosa, inflammatory cell infiltration, and the presence of foreign-body giant cells. Masson’s trichrome staining showed severe fibrosis in the periureteral tissues (Figure 4D), while Pizzolato staining and polarized light microscopy revealed macrocalcifications (Figure 4E,F). Inflammatory cell infiltration and fibrosis, microcalcification, and a loss of urothelium were observed in 13 (92.9%), 8 (57.1%), and 4 (28.6%) patients, respectively.

## 4. Discussion

This study demonstrated that RAUU is an effective and safe treatment for complex US post-URSL. To our knowledge, this is the first study to reveal that most cases exhibited pathological changes, including fibrosis, calcification, and a loss of the urothelium, which may potentially contribute to severe US.

Managing US poses a challenge as surgical options may be limited depending on the etiology, length, and site of US. US may have various etiologies, including congenital ureteropelvic junction obstruction, traumatic or immunological diseases, compression from malignant disorders, retrocaval ureters, ureteral calculi, and previous endourological procedures. Therefore, care must be taken when treating US caused by endourological procedures, particularly considering its iatrogenic nature and the generally low success rates of endoscopic management. We previously reported a significantly lower success rate of endoscopic management for US post-URSL than other etiologies (30% vs. 100%, *p* = 0.004) [6]. The ureteral damage caused by URSL was reportedly associated with ischemic changes, potentially contributing to the failure of endoscopic treatment [18,19]. In this study, 64.2% of patients had undergone previously failed endoscopic interventions. Redo procedures for complex US are usually difficult because of periureteral fibrosis, adhesions, and decreased vascularity. Therefore, in such cases, open surgery, laparoscopic repair, and robot-assisted ureteral reconstruction may be good options with excellent outcomes [20,21,22].

Recent advances in robot-assisted ureteral reconstruction for US have expanded the indications, including ureterostomy, ureteral reimplantation, buccal mucosa graft (BMG) ureteroplasty, and ileal replacement [23,24,25,26,27]. Robotic approaches have a comparable efficacy to open techniques, with some perioperative advantages in terms of blood loss, surgical time, and hospitalization duration [10,28]. Moreover, robotic surgery also has a shorter learning curve for suturing than laparoscopic surgery. RAUU is a simple surgery yet effective technique that involves end-to-end anastomosis of the viable ureter after the damaged ureter has been transected. Hemal et al. retrospectively reviewed 12 RAUU cases for proximal US or retrocaval ureters [29]. However, only a few studies have evaluated the efficacy of RAUU because it requires a watertight, tension-free, and well-vasculated anastomosis, and the indications for RAUU are relatively small. Particularly, surgeons usually opt against RAUU for distal US because of its low success rate due to the tenuous plexiform vessels that supply the distal ureter. The success rate of RAUU in carefully selected patients was >90% in a previous study [25,30,31]. However, the success rate for managing complex US post-URSL in this study was as high as 92.0%, including one distal US case, at a mean follow-up of 12.8 months. The length of US appears to be a significant factor when considering indications for RAUU. A US of <40 mm was considered the preferred choice of ureteroureterostomy [23]. The mean stricture length was not as long as 22.66 mm in this cohort, contributing to a relatively favorable treatment outcome. Furthermore, we found no major perioperative complications (≥grade III) according to the Clavien–Dindo classification, similar to previous reports [25,31]. Therefore, our findings suggest that RAUU plays a role in the management of US post-URSL.

Alternatively, kidney mobilization, ileal ureter replacement, and autotransplantation have been preferred management techniques for a long US of ≥40 mm. Robotic platforms are used for all procedures, and they provide excellent functional outcomes; however, these robotic reconstructions may be technically challenging and associated with substantial morbidity [27,32]. Yang et al. reported that ileal ureter replacement contributed to the associated bowel complications, including urinary tract infection and metabolic acidosis [27]. Breda et al. stated that robot-assisted autotransplantation contributed to renal functional preservation in cases of long ureteral strictures, although the 90-day Clavien–Dindo (≥grade III) complications were as high as 13.8%, including vascular complications [32]. Although we could not directly compare the surgical outcomes between RAUU and other alternative management methods because of their different indications, RAUU has a considerable advantage in terms of fewer complications. Recently, BMG ureteroplasty has been widely applied to long US to overcome the technical complexity and severe complications in robotic ileal ureter replacement and robot-assisted autotransplantation. BMG is used as an onlay flap over the anterior ureteral defect. Lee et al. reported, in their retrospective review of 12 patients who received robotic ureteroplasty with BMG, that it contributed to a satisfactory success rate with low inherent morbidity [33]. Therefore, in the future, robotic ureteroplasty with BMG may be an attractive option in reconstructing the long-segment stricture of the proximal and middle ureters.

Nevertheless, the versatility of the robotic platform in minimally invasive procedures poses specific challenges in identifying precise margins between the stricture and healthy ureteral tissue because the surgeon must rely on visual information due to insufficient tactile feedback. Several approaches have been reported for visually localizing US boundaries. Buffi et al. used a flexible ureteroscope to identify stricture in three of five cases during RAUU [23]. Lee et al. reported the efficacy of an intraluminal ICG injection for the rapid identification of US under NIRF using the Firefly system in a retrospective review of 26 robot-assisted ureteral reconstructions [11]. Notably, we successfully identified the US location by using an illumination catheter in six cases in this study. The IRIS is a new system that facilitates the visualization of the ureter without a contrast agent, such as methylene blue or ICG, by inserting a thin translucent fiber connected to an NIRF source into a ureteral catheter. Interestingly, the brightness can be adjusted, making it useful for patients with fibrosis-induced thickened ureteral walls. To the best of our knowledge, this is the first study to evaluate the efficacy of IRIS during RAUU. Therefore, IRIS provides a promising avenue for enhanced visualization during RAUU procedures.

Some reports on the risk factors of postoperative US post-URSL exist. Sunaryo et al. reported, in a large population-based database of 329,776 patients, that the US rate post-URSL (2.9%) was higher than that of post-shock-wave lithotripsy (1.5%), which may indicate a negative effect of the endoscopic device on stricture formation [3]. Ulvik et al. reported that the use of a ureteral access sheath (UAS), ureteral perforation, and long surgical time were risk factors for postoperative US formation. However, they only discussed the clinical outcomes post-URSL, and only a few reports have evaluated ureteral tissue that underwent US. To the best of our knowledge, this is the first study to examine the histopathology of the transected ureteral tissue leading to US. In this cohort, most patients exhibited inflammatory cell infiltration and fibrosis in the surrounding tissue. Furthermore, important factors in US formation may include the wound-healing response to damage and the subsequent remodeling of the ureteral wall; some inflammatory and wound-healing cytokines are also involved in this process [34]. The inflammatory process subsequent to ureteral damage forms a fibrinous exudate at the site of the tissue injury as part of the immune response, promoting adhesion and, ultimately, stricture formation. Additionally, urine leakage from the ureter contributes to periureteral fibrosis, particularly in the presence of an infection [18]. Patients who underwent previous treatment tended to have severe fibrosis and inflammatory changes surrounding the ureter.

In this study, 57.1% of patients had microcalcifications embedded in the ureteral mucosa. Dretler et al. [35] reported that migrated stone fragments into ureteral mucosa facilitate the formation of “stone granuloma,” where macrophage and foreign-body giant cells could be observed, resulting in the development of US. Specifically, migration to the site of ureteral perforation might be the most severe cause of US. Moreover, the causes of ureteral perforation include a missed shot of the holmium-yttrium aluminum garnet (YAG) laser and UAS insertion into a narrow ureter. Traxer et al. found that 46.5% of patients who underwent URSL with 12/14-F UAS had ureteral wall injuries [36]. When stone dust migrates into areas of the UAS-induced ureteral injury, US may develop in a different area from the stone-impaction site. In this study, 28.6% of the patients exhibited US at a different site from where the previous stone was localized.

Additionally, thermal injuries may negatively affect US formation. Our study revealed that 28.6% of patients had a loss of ureteral mucosa due to damage from laser ablation. Holmium-YAG lasers are considered safe for use in endourological settings because they are completely absorbed by water within 0.4 mm. However, a risk of direct or indirect thermal injuries exists. Direct thermal injury occurs due to the attachment of laser bubbles, while indirect injuries occur when the fluid temperature around the laser increases excessively. Particularly, temperature increases occurred with continuous laser irradiation without irrigation [37]. Furthermore, with the recent introduction of high-power lasers, some studies have reported their efficacy and safety during the procedure, particularly the dusting technique’s clinical benefits [38]. However, the potential risk of laser thermal injury from the improper use of high-power lasers must be considered.

This study has some methodological limitations. First, this descriptive study had a small sample size, potentially limiting the evaluation of the RAUU efficacy. Second, no control group treated with an alternative intervention, such as open or laparoscopic ureteroureterostomy, was included. Therefore, a subsequent larger cohort and comparative studies are warranted to further validate our findings. Nonetheless, this is the first study to focus on US post-URSL and analyze the pathological features of these changes. Consequently, our results highlight the importance of preventing ureteral damage during URSL.

## 5. Conclusions

We evaluated the safety and efficacy of RAUU for US post-URSL. Despite the severe pathological changes observed in the ureter, this technique, which employed end-to-end anastomosis, may be a viable option for US post-URSL in appropriately selected patients. Furthermore, a detailed observation of the pathological findings in the future may lead to the elucidation of the cause of US post-URSL and its prevention.

## Figures and Tables

**Table 1:** Patient and stone characteristics.

*Patient Characteristics*		
age (years)	49.8	(10.2)
sex (%)		
male	10	(71.4)
female	4	(28.6)
laterality (%)		
right	6	(42.9)
left	8	(57.1)
BMI (kg/m^2^)	23.07	(2.11)
***Characteristics of previous urinary calculi***		
location (%)		
UPJ	4	(28.6)
proximal ureter	7	(50.0)
middle ureter	2	(14.3)
distal ureter	1	(7.1)
stone size (mm)	11.85	(11.00, 14.75)
stone volume (mm^2^)	78.11	(72.76, 99.11)
number of previous stone treatment		
SWL	0.00	(0.00, 0.00)
URSL	0.00	(0.00, 0.00)
PCNL	2.00	(1.00, 2.00)
stone analysis (%)		
calcium stone	8	(57.1)
ammonium Acid Urate	1	(7.1)
cystine	1	(7.1)
unknown	4	(28.6)

Mean (SD), median (25% IQR, 75% IQR), and number (%). BMI, body mass index; UPJ, ureteropelvic junction; SWL, shock wave lithotripsy; URSL, ureteroscopic lithotripsy; PCNL, percutaneous nephrolithotomy; SD, standard deviation; IQR, interquartile range.

**Table 2:** Ureteral stricture characteristics.

Stricture Location (%)		
UPJ	3	(21.4)
proximal ureter	7	(50.0)
middle ureter	3	(21.4)
distal ureter	1	(7.1)
deviation from the previous stone site (%)	4	(28.6)
stricture length on radiography (mm)	22.66	(7.38)
prior treatment for stricture (%)		
none	5	(35.7)
dilation	1	(7.1)
endoscopic incision plus dilation	8	(57.1)
frequency of prior treatment for stricture times	1.00	(0.00, 1.00)
preoperative hydronephrosis (%)		
none	0	(0)
mild	0	(0)
moderate	4	(28.6)
severe	10	(71.4)
preoperative drainage (%)		
none	1	(7.1)
ureteral stent	10	(71.4)
percutaneous nephrostomy	3	(21.4)
split renal function of the affected side (%)	29.28	(15.31)

Mean (SD), median (25% IQR, 75% IQR), and number (%). UPJ, ureteropelvic junction; SD, standard deviation; IQR, interquartile range.

**Table 3:** Perioperative variables.

Stricture Identification		
ICG	8	(57.1)
IRIS catheter	6	(42.9)
transected length of the ureter (mm)	26.36	(9.60)
surgery time (min)	205.53	−46.51
console time (min)	164.36	(44.18)
blood loss (mL)	10.00	(0.00, 57.00)
perioperative complication (Clavien–Dindo grade; %)		
0	12	(85.7)
I	1	(7.1)
II	1	(7.1)
≥III	0	(0)
postoperative hydronephrosis after 3 months (%)		
none	2	(14.3)
mild	11	(78.6)
moderate	1	(7.1)
severe	0	
success rate (%)	13	(92.9)
pathological features (%)		
inflammatory cell infiltration	13	(92.9)
fibrosis	13	(92.9)
calcification	8	(57.1)
loss of urothelium	4	(28.6)

Mean (SD), median (25% IQR, 75% IQR), and number (%). ICG, indocyanine green; IRIS, infrared illumination system; SD, standard deviation; IQR, interquartile range.

## Data Availability

The data presented in this study are available upon request from the corresponding author.
